# Comparative effectiveness of different exercise interventions for elderly patients with hip fracture: A systematic review and Bayesian network meta-analysis protocol of randomized controlled trials

**DOI:** 10.1371/journal.pone.0288473

**Published:** 2023-09-07

**Authors:** Rong-jia Pan, Si-Jie Gui, Ting Wang, Fang Nian, Ao-yi Wang, Cai-juan Liu, Zhuo-lan Li, Dan Peng, Gu-qing Zeng

**Affiliations:** 1 School of Nursing, Hengyang Medical School, University of South China, Hengyang, Hunan Province, China; 2 Department of Orthopedics, The Second Xiangya Hospital of Central South University, Changsha, Hunan, China; University of Liege: Universite de Liege, BELGIUM

## Abstract

**Background:**

Exercise intervention (EI) is a promising and economical way for elderly patients with hip fracture, but the evidence regarding effective EIs remains fragmented and controversial, and it is unclear which type of exercise is optimal. The purpose of this Bayesian network meta-analysis (NMA) is to compare and rank the efficacy of various EIs in elderly patients with hip fracture.

**Materials and methods:**

A comprehensive literature search was performed using a systematic approach across various databases including Medline (via PubMed), CINAHL, CNKI, Web of Science, Wan Fang, Embase, VIP, Cochrane Central Register of Controlled Trials and CBM databases. The search encompasses all available records from the inception of each database until December 2022. The Inclusion literature comprises randomized controlled trials that incorporate at least one EI for elderly patients with hip fracture. We will assess the risk of bias of the studies in accordance with the Cochrane Handbook for Systematic Reviews of Interventions, and assess each evidence of outcome quality in accordance with the Grading of Recommendations Assessment, Development and Evaluation framework. The NMA will be performed by STATA 15.0 software and OpenBUGS version 3.2.3. The identification of publication bias will be accomplished through the utilization of a funnel plot. We will rank the EIs effects according to the cumulative ranking probability curve (surface under the cumulative ranking area, SUCRA). The primary outcomes will be hip function in elderly patients, and the secondary outcomes will be activities of daily living, walking capacity and balance ability of elderly patients.

**Trial registration:**

PROSPERO registration number: CRD4202022340737.

## 1 Introduction

Hip fracture is considered as one of the most severe type of fracture due to its elevated rates of high morbidity [1], mortality [2], and disability [3]. More than 87% of hip fracture patients are 65 years old or older, hip fracture has a serious influence on the function of limb, quality of life and increases the burden on caregivers and social substantially because of infirmity and loss of mobility for elderly patients [4]. Many studies have reported that only 40–70% of patients with hip fracture recovered from basic activities of life and only 40–60% of patients recovered to the gait level same as before fracture [5]. With the acceleration of population aging, hip fracture in the elderly has become an increasingly serious public health problem in the world. Therefore, a pressing concern is how to guarantee expeditious restoration of physical function in elderly patients who have undergone hip fracture surgery [6].

There are several ways to promote rapid recovery of patients after hip fracture, mainly including physical therapy and exercise therapy. Exercise interventions (EIs) mainly include aerobic exercise, resistance exercise, muscle strength exercise, balance exercise, vibration exercise and weight-bearing exercise [7, 8]. In recent years, EIs have gradually become an important method to explore healthy outcome in patients with hip fracture. EIs can significantly improve the ability to walk longer distances and are recommended as first-line therapy [9]. Prior exercise trials have reported that EIs could shorten the length of hospital stay, reduce the incidence of complications and promote physical functional recovery in elderly patients with hip fracture [10, 11]. Experimental studies [12] have suggested that EIs could maintain peroxisome proliferator-activated receptor-γ coactivator-1α levels, and inhibit in vivo Forkhead boxO3-induced, which reduced muscle atrophy, maintain muscle function and increased hip stability. Meanwhile, EIs were crucial for the activity of osteoblastic and osteoclastic activities and maintaining the skeletal system, thereby improving hip function. More importantly, exercise as a therapeutic intervention for hip fracture has been recommended by clinical guidelines [13]. As a result of its notable safety, well-proven efficacy and minimal adverse effects, EI has become a widely utilized intervention in the management of hip fractures. Although there are several studies related to EIs after hip fracture, the evidence regarding effective EIs remains fragmented and controversial. Firstly, studies [14] have shown that EIs can improve the activity of daily life (ADL) and walking ability in those who have hip fracture, but Magaziner [15] suggested that ADL and walking ability of patients had not been significantly improved after exercise training. Due to the disparities in the results of randomized controlled trials (RCTs), an updated and comprehensive meta-analysis is urgently required to offer more dependable evidence. Secondly, most of the previous studies have some limitations, such as small sample sizes, low-quality study design and short follow-up periods [16, 17], which to some extent affect the accurate judgment of the efficacy of EIs, and their long-term effects still need to be further confirmed. Thirdly, the single outcome indicator in the previous literature can only reflects the effect of EIs on hip fracture patients in a one-sided way, it impedes the ability of medical personnel to render a comprehensive and conclusive assessment of the effectiveness of EIs, thereby failing to meet the practical demands of clinical practice [18]. Finally, the optimal evidence for EI in hip fracture patients is unclear as traditional meta-analysis is mostly focused on comparisons of single EI [19] and lacks direct comparisons of different EI, this limitation poses a challenge for healthcare professionals in devising the most effective rehabilitation protocol.

Network meta-analysis (NMA) is a new method for comparing direct and indirect evidence that helps researchers gather evidence from multiple RCTs and compares the relative effectiveness of multiple interventions. It overcomes the limitation of traditional pare-wise meta-analysis and ranks the probability of each intervention’s relative efficacy [20, 21]. Our study aims to identify the optimal exercise-based strategy through a Bayesian NMA and provides a reference for policymakers and clinical researchers.

## 2 Materials and methods

In this network meta-analysis, the protocol was based on the guidelines in Cochrane Handbook for Systematic Reviews of Interventions [22]. There was strict adherence to the Preferred Reporting Items for Systematic review and Meta-Analysis Protocols (PRISMA-P) guidelines [23] **(S1 File).** In PROSPERO, this review protocol was registered as CRD4202022340737. In the final report, any observed deviations from the protocol will be documented as a protocol amendment.

### 2.1 Eligibility criteria

We have conducted inclusion and exclusion criteria in the PICOS format. These are described in detail in **Table 1.**

**Table 1:** Inclusion and exclusion criteria.

	Inclusion	Exclusion
Population	(1) Older adults aged 60 years and above; (2) Participants were diagnosed with hip fracture according to X-ray and computed tomography (CT) scan.	Participants were complicated with other diseases, such as dementia.
Intervention	Intervention groups must include one type of exercise (such as Aerobic Exercise, Muscle Strength Exercise, Resistance Exercise, Balance Exercise, Weight-Bearing Exercise, etc.)	(1) Intervention groups include two or more mixed exercise interventions; (2) Intervention groups included other intervention types (eg: pharmacological, nutritional and other interventions)
Comparator	The comparator received no intervention, usual care or other forms of exercise.	The comparator received multicomponent care (eg:health education)
Outcome	The primary outcome includes hip function and the secondary outcome includes ADL, walking capacity and balance ability.	Outcome was incomplete or unavailable.
Study designs	Randomized controlled studies	Non-randomized clinical trials, case reports, reviews or protocols

### 2.2 Data source and search strategy

A systematic search of literature was carried out in China Science and Technology Journal Database (VIP), CINAHL, Web of Science, Cochrane Central Register of Controlled Trials (CENTRAL), China National Knowledge Infrastructure database (CNKI), Embase, Wan Fang database, Medline (via PubMed) and China Biomedical Literature Database (CBM) from their inception to December 2022. The present study will employ the following search terms and their combinations: "hip fractures", "resistance training", "aerobic exercise", "postural balance", "muscle strength", "weight-bearing exercise" and "randomized controlled trial" to conduct a comprehensive search for pertinent literature. The complete search strategies for all databases are shown in the **S2 File**. Additionally, we will manually examine the list of references included in the study and the systematic review or meta-analysis of related studies. CADTH’s Grey Matter Light, Google Scholars, Clinical Trials Registry (clinicaltrials.gov) will be used to search the grey literature for a thorough search. Subsequently, we intend to engage with specialists in orthopedics, sports medicine, and rehabilitation to scrutinize any overlooked articles through the suggested search methodology. There are no limitations imposed on the publication language, country of study and date of included studies in our study.

### 2.3 Study selection

**Fig 1:**
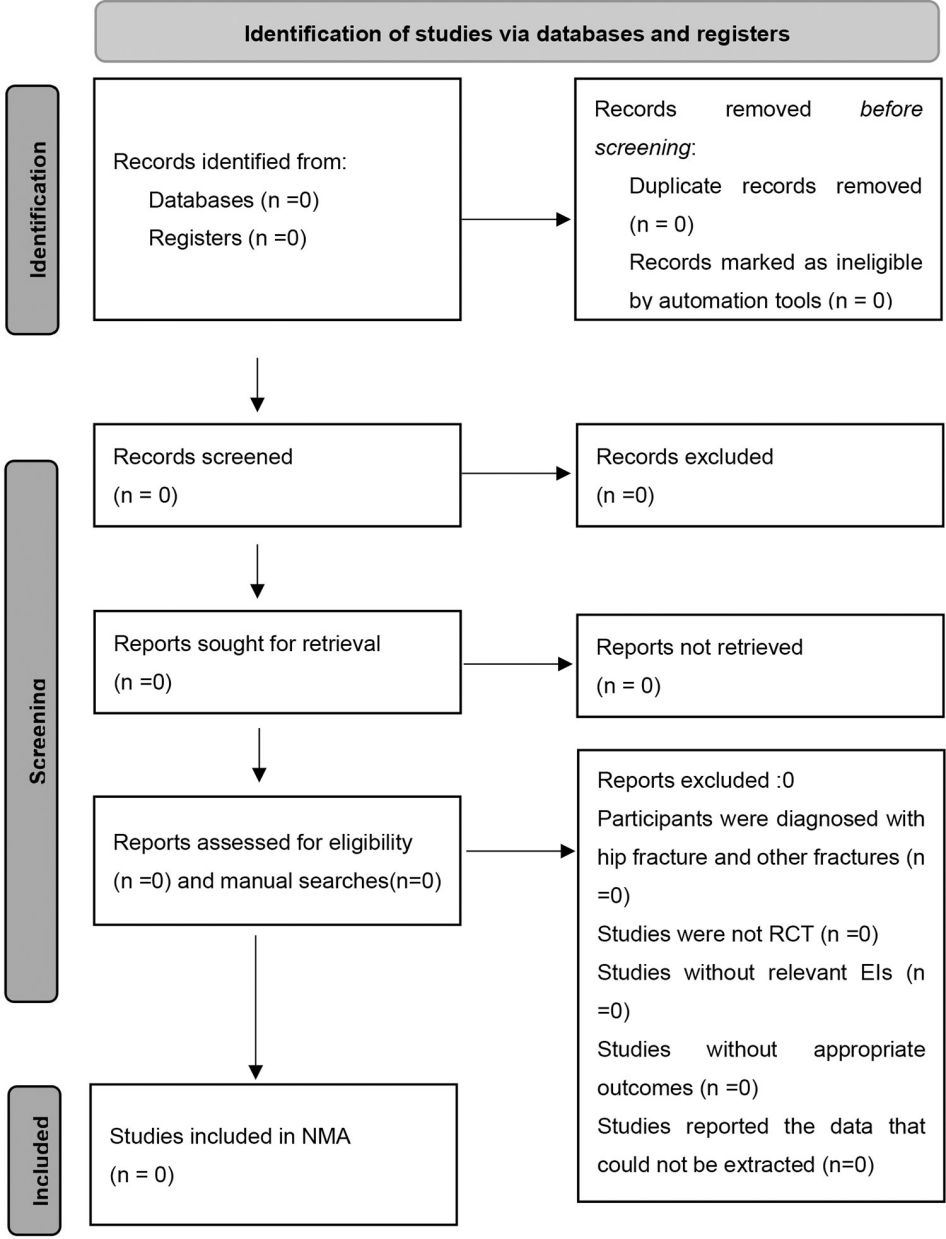
Selection of studies for inclusion. EI = exercise-based intervention; NMA = Network meta-analysis; RCT = randomized controlled trial.

Two reviewers will use Endnote X9 software to screen all initial search results independently. Relevance screening includes third stages: (1) Removing duplicate articles; (2) Articles will be screened by reading title and abstract; (3) Articles will be screened by reading full-text. The studies will be reviewed by two independent reviewers and disagreements concerning inclusion or exclusion of studies will be resolved by consensus. Cohen’s kappa coefficient will be used to assess the agreement between the reviewers for inclusion/exclusion of studies [24]. The final study inclusion will be presented as a flow chart, using a PRISMA flow diagram **(Fig 1)**.

### 2.4 Data extraction

Two authors will use a standardized extraction form to extract the data from the included studies independently. Any disagreements for data extraction of studies will be resolved by the third reviewer. When data are presented as a figure, in this case, we will use image digitizing software Engauge Digitizer software (version 4.1) (http://markummitchell.github.io/engauge-digitizer/) to obtain and extract the values from figures. Using the data extraction form, the authors will extract data from the included studies **(S3 File)**: first author, mean age, country, exercise type (frequency, intensity, duration), year of publication, sample size, comparison, intervention site, outcome measures reported. If there were incomplete demographic data in literature, such as age, sex, and duration of hospitalization, we still chose to include this article, since it will not affect the research results. Where data are missing from the published report of a study, we will attempt to contact the author to obtain the data and clarify any uncertainties. In addition, we will perform sensitivity analysis where possible if the missing data posed a high risk of bias. In addition, for those studies not reporting standard deviation (SD), the SD will be acquired through the utilization of the techniques recommended in Section 7.7.3 of the Cochrane Handbook for Systematic Reviews of Interventions [25]. For continuou`s data, we will estimate missing SDs by standard errors, confidence interval, t-value, and P-value for single or combined conversion. Otherwise, we will not have undertaken any imputations or use any statistical methods to impute missing data.

### 2.5 Risk of bias assessment and GRADE

We will utilize the Revised Cochrane risk-of-bias tool for randomized trials (ROB 2.0) to gauge the risk in the findings of included studies. It includes five domains of bias: (1) randomization process, (2) deviations from the intended interventions, (3) missing outcome data, (4) measurement of the outcome, (5) selection of the reported result. Each domain contains signaling questions, which can be scored with “Yes,” “Probably yes,” “Probably no,” “No,” or “No information” [26]. Two authors will independently evaluate the ROB for each included study. The third author will resolve disagreement by arbitration or by consensus. Publication bias will be presented by funnel plots and assessed by the Egger test. Additionally, in accordance with the Grading of Recommendations Assessment, Development and Evaluation (GRADE) framework, two reviewers will assess the strength and reliability of evidence for the primary and secondary outcomes individually [27].

### 2.6 Statistical analysis

#### 2.6.1 Pairwise meta-analysis

We will assess the heterogeneity among the studies with the Cochran Q test (χ^2^ test for heterogeneity) and *I*^2^ statistic. The percentage of total heterogeneity to total variability will be quantified by *I*^2^ statistic [28]. When significant heterogeneity (*p* < 0.05, *I*^2^ ⩾ 50%) is observed, it can be indicated that there is a high heterogeneity and the random effects model will be applied in the meta-analysis. When there is no statistically significant heterogeneity (*p* < 0.05, *I*^2^ < 50%), sensitivity analysis will be planned to test the impact of choosing an a priori random effect model on the results by running a second time the analysis using a fixed effect model. For continuous variables, if research outcome indicators have the same unit measurements, weighted mean difference (WMD) with 95% confidence interval will be used; if outcome measures are different units or measures, standardized mean difference (SMD) with 95% confidence interval will be chosen as pooled statistic. Besides, SMD will be used as the effect size when there is a large difference in mean or mean deviation among the included studies [29, 30].

#### 2.6.2 Network meta-analysis

Before performing data synthesizing, we will assess the transitivity assumption and consistency assumption. We will collect detailed information related to patient characteristics, interventions, primary outcome and secondary outcomes to assess the transitivity assumption for NMA [31]. Maximum likelihood and Bayesian inference will be used to analyze indirect comparisons of different EIs [32]. A network relation graph is a special tool for displaying the evidence [33]. In the NMA graph, (i) the size of the nodes reflects how many elderly patients with hip fracture were assigned to the corresponding group across all trials, and (ii) the lines indicate direct comparisons between the two EIs, the thickness of the connecting line is proportional to the number of direct comparisons. Subsequently, we will verify the homogeneity and consistency assumptions between direct evidence and indirect evidence by using node splitting and Bland Altman’s method [34]. For all Bayesian formulations, three Markov chain Monte Carlo (MCMC) simulations will run for 1,000,000 generations, sampling every 5000 generations. At the same time, we will discard 20,000 as burn-in to make the output more manageable, trace plots and Brooks-Gelman-Rubin statistics will be used to ensure convergence [35, 36]. We will summarize and report the probability of each EIs being the best intervention using the surface under the cumulative ranking (SUCRA) curve. Higher values indicate that EI is more likely to be effective than other exercises or usual care for patients with hip fracture [37]. We will compare and rank the efficacy of various EIs in elderly patients with hip fracture by establish a Bayesian network meta-analysis random effect model, the effect size of each EI will be expressed as mean differences (MD) and 95% credible interval (95%CrI). To assess the presence of publication bias in NMA, we will generate a network funnel plot and check its symmetry, and asymmetrical funnel plots will be subjected to an Egger test [38]. Two reviewers plan to assess the quality of the evidence and strength of recommendation for each outcome using the GRADE approach. We will adopt STATA software 15.0 (StataCorp, College Station, TX, USA) to present the results and graphs of the NMA [39] and will perform NMA within a Bayesian framework via the Markov chain Monte Carlo method in OpenBUGS software (version 3.2.3) (http://www.openbugs.info/w.cgi/Downloads) [40].

### 2.7 Sensitivity analyses and subgroup analysis

We will conduct sensitivity analysis by modifying inclusion criteria for controversial literature, using different statistical methods, and excluding low-quality literature to determine the robustness of the results [41]. To detect other potential sources of heterogeneity, meta-regression analysis and subgroup analysis will be performed in our analysis. We will carry out subgroup analyses according to duration, intensity and frequency of EIs, to examine the influence of the different moderator variables on the obtained effect sizes. If the data are numerical variable, we will analyze it by meta-regression.

### 2.8 Ethics and dissemination

No formal research ethics approval is required. The results will be disseminated to a peer-reviewed journal for publication.

## 3 Strengths and limitations of this study

The present study has the following strengths. First, our study was the first time to use NMA to verify the effectiveness of different EIs after hip fracture. The language and publication date were not restricted in our study, our literature search strategy used multiple databases to identify as many studies as possible, the sample size was large and representative.

The limitations of our study should be discussed. There is no standardized definition of nursing measures for the control group in the included literature, which may lead to uncertainty in the results of the study. Besides, in the included literature, due to differences in the timing of EIs, the long-term efficacy of different exercise types is unclear for elderly patients with hip fracture.

## Supporting information

S1 File Research checklist—PRISMA-P (Preferred Reporting Items for Systematic review and Meta-Analysis Protocols) 2015 checklist. (DOCX)

S2 File The initial search strategy for all databases. (DOCX)

S3 File Data abstraction form for analysis. (DOCX)
