# Supplementary material for: Comparative effectiveness of different exercise interventions for elderly patients with hip fracture: A systematic review and Bayesian network meta-analysis protocol of randomized controlled trials
Source: PLoS One. 2023 Sep 7;18(9):e0288473. doi: 10.1371/journal.pone.0288473 (PMC10484429; doi:10.1371/journal.pone.0288473)
Supplement: S2 File — (DOCX) [file pone.0288473.s002.docx]

**File 2 The initial search strategy for all databases**

Table 1 Search strategy in PubMed

| Step | Search strategy |
| --- | --- |
| #1 | (hip fractures[MeSH Terms]) OR (intertrochanteric fracture*[Title/Abstract]) OR (subtrochanteric fracture*[Title/Abstract]) OR (femoral neck fracture*[Title/Abstract]) OR (trochanteric fracture*[Title/Abstract]) OR (femur neck fracture*[Title/Abstract]) OR (hip fracture[Title/Abstract])) |
| #2 | ((Mind-Body Therapies[MeSH Terms]) OR (Breathing Exercises[MeSH Terms]) OR (Autogenic Training[MeSH Terms]) OR (Relaxation Therapy[MeSH Terms]) OR (Exercise[MeSH Terms]) OR (Rehabilitation[MeSH Terms]) OR (sports[MeSH Terms]) OR (Exercise Therapy[MeSH Terms]) OR (movement[MeSH Terms]) OR (early ambulation[MeSH Terms]) OR (Muscle Strength[MeSH Terms]) OR (resistance training[MeSH Terms]) OR (Circuit-Based Exercise[MeSH Terms]) OR (postural balance[MeSH Terms]) OR (occupational therapy[MeSH Terms]) OR (vibration training[Title/Abstract]) OR (Mind-Body Therapy[Title/Abstract]) OR (Mind-Body Medicine[Title/Abstract]) OR (Mind Body Medicine[Title/Abstract]) OR (Qigong[Title/Abstract]) OR (Tai Ji[Title/Abstract]) OR (Yoga[Title/Abstract]) OR (Tai Chi[Title/Abstract]) OR (Tai Ji Quan[Title/Abstract]) OR (Tai Chi Chuan[Title/Abstract]) OR (Baduanjin[Title/Abstract]) OR (Wuqinxi[Title/Abstract]) OR (Yijinjing[Title/Abstract]) OR (Physical Activit*[Title/Abstract]) OR (Physical Exercise*[Title/Abstract]) OR (Acute Exercise*[Title/Abstract]) OR (Isometric Exercise*[Title/Abstract]) OR (Aerobic Exercise*[Title/Abstract]) OR (Exercise Training*[Title/Abstract]) OR (Remedial Exercise*[Title/Abstract]) OR (Rehabilitation Exercise*[Title/Abstract]) OR (walking[Title/Abstract]) OR (training[Title/Abstract]) OR (retraining[Title/Abstract]) OR (mobili*[Title/Abstract]) OR (stepping[Title/Abstract]) OR (fall prevention exercise[Title/Abstract]) OR (foot taps[Title/Abstract]) OR (step up[Title/Abstract]) OR (gait[Title/Abstract]) OR (locomotion[Title/Abstract]) OR (motor activity[Title/Abstract]) OR (physio therap*[Title/Abstract]) OR (physical therap*[Title/Abstract]) OR (endurance[Title/Abstract]) OR (strength*[Title/Abstract]) OR (functional exercise*[Title/Abstract]) OR (ADL training[Title/Abstract]) OR (strength-promoting exercise*[Title/Abstract]) OR (stretching exercise*[Title/Abstract]) OR (standing exercise*[Title/Abstract]) OR (fexibility exercise*[Title/Abstract]) OR (Therapeutic Relaxation[Title/Abstract]) OR (Relaxation Technique[Title/Abstract]) OR (Relaxation Technic*[Title/Abstract]) OR (Nature Therap*[Title/Abstract]) OR (Ecotherap*[Title/Abstract]) OR (home rehabilitation[Title/Abstract]) OR (outpatient rehabilitation[Title/Abstract]) OR (home-based[Title/Abstract]) OR (Community exercise*[Title/Abstract]) OR (Progressive Relaxation[Title/Abstract]) OR (Strength Training*[Title/Abstract]) OR (Weight Lifting Strengthening Program*[Title/Abstract]) OR (Weight Lifting Exercise Program*[Title/Abstract]) OR (Weight-Bearing Strengthening Program*[Title/Abstract]) OR (Weight Bearing Exercise Program*[Title/Abstract]) OR (elastic tube[Title/Abstract]) OR (eccentric[Title/Abstract]) OR (concentric[Title/Abstract]) OR (pulleys[Title/Abstract]) OR (Habilitation[Title/Abstract]) OR (Circuit Based Exercise*[Title/Abstract]) OR (Circuit-Based Exercises[Title/Abstract]) OR (Circuit Training[Title/Abstract]) OR (balance exercise*[Title/Abstract]) OR (balance training[Title/Abstract]) OR (standing on one leg[Title/Abstract]) OR (balance equipment[Title/Abstract])) |
| #3 | (randomized controlled trial [pt]) OR (randomi* [tiab]) OR (randomized controlled trial [pt]) OR (controlled clinical trial [pt]) OR (randomized [tiab]) OR (randomly [tiab]) OR (trial [tiab]) OR (groups [tiab])) |
| #4 | #1 AND #2 AND #3 |

Table 2 Search strategy in Web of science

| Step | Search strategy |
| --- | --- |
| #1 | TS=(intertrochanteric fracture*) OR TS=(subtrochanteric fracture*) OR TS=(femoral neck fracture*) OR TS=(trochanteric fracture*) OR TS=(femur neck fracture*) OR TS=(hip fracture*) |
| #2 | ((TS=(vibration training) OR TS=(Mind-Body Therapy) OR TS=(Mind-Body Medicine) OR TS=(Qigong) OR TS=(Tai Ji) OR TS=(Yoga) OR TS=(Tai Chi) OR TS=(Tai Ji Quan) OR TS=(Tai Chi Chuan) OR TS=(Baduanjin) OR TS=(Wuqinxi) OR TS=(Yijinjing) OR TS=(Physical Activit*) OR TS=(Physical Exercise*) OR TS=(Acute Exercise*) OR TS=(Isometric Exercise*) OR TS=(Aerobic Exercise*) OR TS=(Exercise Training*) OR TS=(Remedial Exercise*) OR TS=(Rehabilitation Exercise*) OR TS=(walking)) OR TS=(training) OR TS=(retraining) OR TS=(mobili*) OR TS=(stepping) OR TS=(fall prevention exercise) OR TS=(foot taps) OR TS=(step up) OR TS=(gait) OR TS=(locomotion) OR TS=(motor activity) OR TS=(physio therap*) OR TS=(physical therap*) OR TS=(endurance) OR TS=(strength*) OR TS=(functional exercise*) OR TS=(ADL training) OR TS=(strength-promoting exercise*) OR TS=(stretching exercise*) OR TS=(standing exercise*) OR TS=(fexibility exercise*) OR TS=(Therapeutic Relaxation) OR TS=(Relaxation Technique) OR TS=(Relaxation Technic*) OR TS=(Nature Therap*) OR TS=(Ecotherap*) OR TS=(home rehabilitation) OR TS=(outpatient rehabilitation) OR TS=(home-based) OR TS=(Community exercise*) OR TS=(Progressive Relaxation) OR TS=(Strength Training*) OR TS=(Weight Lifting Strengthening Program*) OR TS=(Weight Lifting Exercise Program*) OR TS=(Weight-Bearing Strengthening Program*) OR TS=(Weight Bearing Exercise Program*) OR TS=(elastic tube) OR TS=(eccentric) OR TS=(concentric) OR TS=(pulleys) OR TS=(Habilitation) OR TS=(Circuit Based Exercise*) OR TS=(Circuit-Based Exercises) OR TS=(Circuit Training) OR TS=(balance exercise*) OR TS=(balance training) OR TS=(standing on one leg) OR TS=(balance equipment) OR TS=(Mind-Body Therapies) OR TS=(Breathing Exercises) OR TS=(Autogenic Training) OR TS=(Relaxation Therapy) OR TS=(Exercise) OR TS=(Rehabilitation) OR TS=(sports) OR TS=(Exercise Therapy) OR TS=(movement) OR TS=(early ambulation) OR TS=(Muscle Strength) OR TS=(resistance training) OR TS=(Circuit-Based Exercise) OR TS=(postural balance) OR TS=(occupational therapy)) |
| #3 | TS= clinical trial* OR TS=research design OR TS=comparative stud* OR TS=evaluation stud* OR TS=controlled trial* OR TS=follow-up stud* OR TS=prospective stud* OR TS=random* OR TS=placebo* OR TS=(single blind*) OR TS=(double blind*) |
| #4 | #1 AND #2 AND #3 |

Table 3 Search strategy in Embase

| Step | Search strategy |
| --- | --- |
| #1 | 'intertrochanteric fracture*':ab,ti OR 'subtrochanteric fracture*':ab,ti OR 'femoral neck fracture':ab,ti OR 'trochanteric fracture*':ab,ti OR 'fracture of hip':ab,ti OR 'femur neck fracture*':ab,ti OR 'fractures, subtrochanteric':ab,ti OR 'fractures, intertrochanteric':ab,ti OR 'fractures, trochanteric':ab,ti OR 'fractures, hip':ab,ti OR 'hip fractures':ab,ti OR 'femoral neck fractures':ab,ti |
| #2 | 'vibration training':ab,ti OR 'mind-body therapy':ab,ti OR 'mind-body medicine':ab,ti OR 'mind body medicine':ab,ti OR 'qigong':ab,ti OR 'tai ji':ab,ti OR 'yoga':ab,ti OR 'tai chi':ab,ti OR 'tai ji quan':ab,ti OR 'tai chi chuan':ab,ti OR 'baduanjin':ab,ti OR 'wuqinxi':ab,ti OR 'yijinjing':ab,ti OR 'physical activit*':ab,ti OR 'physical exercise*':ab,ti OR 'acute exercise*':ab,ti OR 'isometric exercise*':ab,ti OR 'aerobic exercise*':ab,ti OR 'exercise training*':ab,ti OR 'remedial exercise*':ab,ti OR 'rehabilitation exercise*':ab,ti OR 'walking':ab,ti OR 'retraining or mobili*':ab,ti OR 'stepping':ab,ti OR 'fall prevention exercise':ab,ti OR 'foot taps':ab,ti OR 'step up':ab,ti OR 'gait':ab,ti OR 'locomotion':ab,ti OR 'motor activity':ab,ti OR 'physio therap*':ab,ti OR 'physical therap*':ab,ti OR 'endurance':ab,ti OR 'strength*':ab,ti OR 'functional exercise*':ab,ti OR 'ADL training':ab,ti OR 'strength-promoting exercise*':ab,ti OR 'stretching exercise*':ab,ti OR 'standing exercise*':ab,ti OR 'fexibility exercise*':ab,ti OR 'therapeutic relaxation':ab,ti OR 'relaxation technique':ab,ti OR 'relaxation technic*':ab,ti OR 'nature therap*':ab,ti OR 'ecotherap*':ab,ti OR 'home rehabilitation':ab,ti OR 'outpatient rehabilitation':ab,ti OR 'home-based':ab,ti OR 'community exercise*':ab,ti OR 'progressive relaxation':ab,ti OR 'strength training*':ab,ti  OR 'weight lifting strengthening program*':ab,ti OR 'weight lifting exercise program*':ab,ti OR 'weight-bearing strengthening program*':ab,ti OR 'weight bearing exercise program*':ab,ti OR 'elastic tube':ab,ti OR 'eccentric':ab,ti OR 'concentric':ab,ti OR 'pulleys':ab,ti OR 'habilitation':ab,ti OR 'circuit based exercise*':ab,ti OR 'circuit-based exercises':ab,ti OR 'circuit training':ab,ti OR 'balance exercise*':ab,ti OR 'balance training':ab,ti OR 'standing on one leg':ab,ti OR 'balance equipment':ab,ti OR 'mind-body therapies':ab,ti OR 'breathing exercises':ab,ti OR 'autogenic training':ab,ti OR 'relaxation therapy':ab,ti OR 'exercise therapy':ab,ti OR 'movement':ab,ti OR 'early ambulation':ab,ti OR  'muscle strength':ab,ti OR 'resistance training':ab,ti OR 'circuit-based exercise':ab,ti OR 'postural balance':ab,ti OR 'occupational therapy':ab,ti |
| #3 | ('crossover procedure':de OR 'double-blind procedure':de OR 'randomized controlled trial':de) AND or  AND 'single-blind procedure':de OR (random*:de,ab,ti AND or :de,ab,ti AND factorial*:de,ab,ti) OR crossover*:de,ab,ti OR ((cross NEXT/1 over*):de,ab,ti) OR placebo*:de,ab,ti OR ((doubl* NEAR/1 blind*):de,ab,ti) OR ((singl* NEAR/1 blind*):de,ab,ti) OR assign*:de,ab,ti OR allocat*:de,ab,ti OR volunteer*:de,ab,ti |
| #4 | 'hip fracture'/exp |
| #5 | #1 AND #4 |
| #6 | 'training'/exp |
| #7 | 'sport'/exp |
| #8 | 'exercise'/exp |
| #9 | 'rehabilitation'/exp |
| #10 | #2 OR #6 OR #7 OR #8 OR #9 |
| #11 | #3 AND #5 AND #10 |

Table 4 Search strategy in Cochrane

| Step | Search strategy |
| --- | --- |
| #1 | MeSH descriptor: [Hip Fractures] explode all trees |
| #2 | (intertrochanteric fracture*):ti,ab,kw OR (subtrochanteric fracture*):ti,ab,kw OR (femoral neck fracture*):ti,ab,kw OR (trochanteric fracture*):ti,ab,kw OR (femur neck fracture*):ti,ab,kw (Word variations have been searched) |
| #3 | #1 OR #2 |
| #4 | MeSH descriptor: [Exercise] explode all trees |
| #5 | MeSH descriptor: [Sports] explode all trees |
| #6 | MeSH descriptor: [Teaching] explode all trees |
| #7 | MeSH descriptor: [Rehabilitation] explode all trees |
| #8 | (vibration training):ti,ab,kw OR (Mind-Body Therapy):ti,ab,kw OR (Mind-Body Medicine):ti,ab,kw OR (Qigong):ti,ab,kw OR (Tai Ji):ti,ab,kw (Word variations have been searched) |
| #9 | (Yoga):ti,ab,kw OR (Tai Chi):ti,ab,kw OR (Tai Ji Quan):ti,ab,kw OR (Tai Chi Chuan):ti,ab,kw OR (Baduanjin):ti,ab,kw (Word variations have been searched) |
| #10 | (Wuqinxi):ti,ab,kw OR (Yijinjing):ti,ab,kw OR (Physical Activit*):ti,ab,kw OR (Physical Exercise*):ti,ab,kw OR (Acute Exercise*):ti,ab,kw (Word variations have been searched) |
| #11 | (Isometric Exercise*):ti,ab,kw OR (Aerobic Exercise*):ti,ab,kw OR (Exercise Training*):ti,ab,kw OR (Remedial Exercise*):ti,ab,kw OR (Rehabilitation Exercise*):ti,ab,kw (Word variations have been searched) |
| #12 | (walking):ti,ab,kw OR (mobili*):ti,ab,kw OR (stepping):ti,ab,kw OR (fall prevention exercise):ti,ab,kw OR (gait):ti,ab,kw (Word variations have been searched) |
| #13 | (locomotion):ti,ab,kw OR (motor activity):ti,ab,kw OR (physio therap*):ti,ab,kw OR (physical therap*):ti,ab,kw OR (endurance):ti,ab,kw (Word variations have been searched) |
| #14 | (strength*):ti,ab,kw OR (functional exercise*):ti,ab,kw OR (ADL training):ti,ab,kw OR (strength-promoting exercise*):ti,ab,kw OR (stretching exercise*):ti,ab,kw (Word variations have been searched) |
| #15 | (standing exercise*):ti,ab,kw OR (fexibility exercise*):ti,ab,kw OR (Therapeutic Relaxation):ti,ab,kw OR (home rehabilitation):ti,ab,kw OR (outpatient rehabilitation):ti,ab,kw (Word variations have been searched) |
| #16 | (home-based):ti,ab,kw OR (Community exercise*):ti,ab,kw OR (Progressive Relaxation):ti,ab,kw OR (Strength Training*):ti,ab,kw OR (Weight Bearing):ti,ab,kw (Word variations have been searched) |
| #17 | (Circuit Based Exercise*):ti,ab,kw OR (Circuit Training):ti,ab,kw OR (balance exercise*):ti,ab,kw OR (balance training):ti,ab,kw OR (Mind-Body Therapies):ti,ab,kw (Word variations have been searched) |
| #18 | (Breathing Exercises):ti,ab,kw OR (Autogenic Training):ti,ab,kw OR (Relaxation Therapy):ti,ab,kw OR (Exercise Therapy):ti,ab,kw OR (movement):ti,ab,kw (Word variations have been searched) |
| #19 | (early ambulation):ti,ab,kw OR (Muscle Strength):ti,ab,kw OR (resistance training):ti,ab,kw OR (Circuit-Based Exercise):ti,ab,kw OR (postural balance):ti,ab,kw (Word variations have been searched) |
| #20 | (occupational therapy):ti,ab,kw (Word variations have been searched) |
| #21 | #4 OR #5 OR #6 OR #7 OR #8 OR #9 OR #10 OR #11 OR #12 OR #13 OR #14 OR #15 OR #16 OR #17 OR #18 OR #19 OR #20 |
| #22 | #3 AND #21 |

Table 5 Search strategy in CINAHL

| Step | Search strategy |
| --- | --- |
| #1 | (hip fractures) OR (intertrochanteric fracture*) OR (subtrochanteric fracture*) OR (femoral neck fracture*) OR (trochanteric fracture*) OR (femur neck fracture*) OR (hip fracture)) |
| #2 | ((Mind-Body Therapies) OR (Breathing Exercises) OR (Autogenic Training) OR (Relaxation Therapy) OR (Exercise) OR (Rehabilitation) OR (sports) OR (Exercise Therapy) OR (movement) OR (early ambulation) OR (Muscle Strength) OR (resistance training) OR (Circuit-Based Exercise) OR (postural balance) OR (occupational therapy) OR (vibration training) OR (Mind-Body Therapy) OR (Mind-Body Medicine) OR (Mind Body Medicine) OR (Qigong) OR (Tai Ji) OR (Yoga) OR (Tai Chi) OR (Tai Ji Quan) OR (Tai Chi Chuan) OR (Baduanjin) OR (Wuqinxi) OR (Yijinjing) OR (Physical Activit*) OR (Physical Exercise*) OR (Acute Exercise*) OR (Isometric Exercise*) OR (Aerobic Exercise*) OR (Exercise Training*) OR (Remedial Exercise*) OR (Rehabilitation Exercise*) OR (walking) OR (training) OR (retraining) OR (mobili*) OR (stepping) OR (fall prevention exercise) OR (foot taps) OR (step up) OR (gait) OR (locomotion) OR (motor activity) OR (physio therap*) OR (physical therap*) OR (endurance) OR (strength*) OR (functional exercise*) OR (ADL training) OR (strength-promoting exercise*) OR (stretching exercise*) OR (standing exercise*) OR (fexibility exercise*) OR (Therapeutic Relaxation) OR (Relaxation Technique) OR (Relaxation Technic*) OR (Nature Therap*) OR (Ecotherap*) OR (home rehabilitation) OR (outpatient rehabilitation) OR (home-based) OR (Community exercise*) OR (Progressive Relaxation) OR (Strength Training*) OR (Weight Lifting Strengthening Program*) OR (Weight Lifting Exercise Program*) OR (Weight-Bearing Strengthening Program*) OR (Weight Bearing Exercise Program*) OR (elastic tube) OR (eccentric) OR (concentric) OR (pulleys) OR (Habilitation) OR (Circuit Based Exercise*) OR (Circuit-Based Exercises) OR (Circuit Training) OR (balance exercise*) OR (balance training) OR (standing on one leg) OR (balance equipment)) |
| #3 | (randomized controlled trial) OR (randomi*) OR (randomized controlled trial) OR (controlled clinical trial) OR (randomized) OR (randomly) OR (trial) OR (groups)) |
| #4 | #1 AND #2 AND #3 |

Table 6 Search strategy in CNKI

| Step | Search strategy |
| --- | --- |
| #1 | SU=(hip fracture+hip joint fracture+fracture of femoral neck+intertrochanteric fracture+femoral subtrochanteric fracture+hip fragility fracture+osteoporosis fracture) |
| #2 | SU=(motion+movement+locomotion+sport+kinesis+exercise+training+physical training+physical function+rehabilitation+resistance+balance+oscillation+vibration+  relaxation+weight-bearing+tolerance+endurance+capacity+aerobic+tai chi  +yoga+qigong+baduanjin+eight-sectioned exercise+eight-section brocade+  five mimic-animal+changing tendon+yijinjing+walking+recovery of joint+muscle strength+physical therapy+athletic sport+activity+strength training+power training+physical strength+task-oriented training+body exercise+functional state+occupational therapy) |
| #3 | SU=(randomized+clinical experiment+clinical trial+application+utilization+influence  +impact+affect+result+effect+curative importance+curative effectiveness+curative efficacy+intervention+observation+analysis+RCT+CCT) |
| #4 | #1 AND #2 AND #3 |

Table 7 Search strategy in WanFang

| Step | Search strategy |
| --- | --- |
| #1 | Title or Keywords:"hip fracture" or "hip joint fracture" or "fracture of femoral neck" or "intertrochanteric fracture" or "femoral subtrochanteric fracture" or "hip fragility fracture" or "osteoporosis fracture" |
| #2 | Title or Keywords:"motion" or "movement" or "locomotion" or "sport" or "kinesis" or "exercise" or "training" or "physical training" or "physical function" or "rehabilitation" or "resistance" or "balance" or "oscillation" or "vibration or relaxation" or "weight-bearing" or "tolerance" or "endurance" or "capacity" or "aerobic" or "tai chi" or "yoga" or "qigong" or "baduanjin" or "eight-sectioned exercise" or "eight-section brocade" or "five mimic-animal" or "changing tendon" or "yijinjing" or "walking" or "recovery of joint" or "muscle strength" or "physical therapy" or "athletic sport" or "activity" or "strength training" or "power training" or "physical strength" or "task-oriented training" or "body exercise" or "functional state" or "occupational therapy" |
| #3 | Title or Keywords:"randomized" or "clinical experiment" or "clinical trial" or "application" or "utilization" or "influence" or "impact" or "affect" or "result" or "effect" or "curative importance" or "curative effectiveness" or "curative efficacy" or "intervention" or "observation" or "analysis" or "RCT" or "CCT" |
| #4 | #1 AND #2 AND #3 |

Table 8 Search strategy in VIP

| Step | Search strategy |
| --- | --- |
| #1 | M=("hip fracture" or "hip joint fracture" or "fracture of femoral neck" or "intertrochanteric fracture" or "femoral subtrochanteric fracture" or "hip fragility fracture" or "osteoporosis fracture") |
| #2 | M=("motion" or "movement" or "locomotion" or "sport" or "kinesis" or "exercise" or "training" or "physical training" or "physical function" or "rehabilitation" or "resistance" or "balance" or "oscillation" or "vibration or relaxation" or "weight-bearing" or "tolerance" or "endurance" or "capacity" or "aerobic" or "tai chi" or "yoga" or "qigong" or "baduanjin" or "eight-sectioned exercise" or "eight-section brocade" or "five mimic-animal" or "changing tendon" or "yijinjing" or "walking" or "recovery of joint" or "muscle strength" or "physical therapy" or "athletic sport" or "activity" or "strength training" or "power training" or "physical strength" or "task-oriented training" or "body exercise" or "functional state" or "occupational therapy") |
| #3 | M=("randomized" or "clinical experiment" or "clinical trial" or "application" or "utilization" or "influence" or "impact" or "affect" or "result" or "effect" or "curative importance" or "curative effectiveness" or "curative efficacy" or "intervention" or "observation" or "analysis" or "RCT" or "CCT") |
| #4 | #1 AND #2 AND #3 |

Table 9 Search strategy in CBM

| Step | Search strategy |
| --- | --- |
| #1 | Theme:(hip fracture OR hip joint fracture OR fracture of femoral neck OR intertrochanteric fracture OR femoral subtrochanteric fracture OR hip fragility fracture OR osteoporosis fracture) |
| #2 | Theme:(motion OR movement OR locomotion OR sport OR kinesis OR exercise OR training OR physical training OR physical function OR rehabilitation OR resistance OR balance OR oscillation OR vibration OR relaxation OR weight-bearing OR tolerance OR endurance OR capacity OR aerobic OR tai chi OR yoga OR qigong OR baduanjin OR eight-sectioned exercise OR eight-section brocade OR five mimic-animal OR changing tendon OR yijinjing OR walking OR recovery of joint OR muscle strength OR physical therapy OR athletic sport OR activity OR strength training OR power training OR physical strength OR task-oriented training OR body exercise OR functional state OR occupational therapy) |
| #3 | Theme:(randomized OR clinical experiment OR clinical trial OR application OR utilization OR influence OR impact OR affect OR result OR effect OR curative importance OR curative effectiveness OR curative efficacy OR intervention OR observation OR analysis OR RCT OR CCT) |
| #4 | #1 AND #2 AND #3 |
