# Supplementary material for: Comparative effectiveness of different exercise interventions for elderly patients with hip fracture: A systematic review and Bayesian network meta-analysis protocol of randomized controlled trials
Source: PLoS One. 2023 Sep 7;18(9):e0288473. doi: 10.1371/journal.pone.0288473 (PMC10484429; doi:10.1371/journal.pone.0288473)
Supplement: S3 File — (DOCX) [file pone.0288473.s003.docx]

**File 3 Data abstraction form for analysis**

| First author,  year | Country | Gender | | Sample size | | Mean age | | Intervention site | Exercise type | | | | Comparison  measure | Outcome measures reported |
| --- | --- | --- | --- | --- | --- | --- | --- | --- | --- | --- | --- | --- | --- | --- |
|  |  | Male  (EG vs CG) | Female  (EG vs CG) | EG | CG | EG | CG |  | Type | Frequency | Intensity | Duration |  |  |
|  |  |  |  |  |  |  |  |  |  |  |  |  |  |  |
|  |  |  |  |  |  |  |  |  |  |  |  |  |  |  |
|  |  |  |  |  |  |  |  |  |  |  |  |  |  |  |

Table 1 Characteristics of included studies

CG,control group; EG,experimental group
